# Spontaneous mesenteric hematoma occurring during antithrombotic therapy and responsive to surgical treatment: a case report

**DOI:** 10.1186/s40792-024-01993-9

**Published:** 2024-08-16

**Authors:** Meiko Aoki, Hisamichi Yoshii, Rika Fujino, Hideki Izumi, Masaya Mukai, Hiroyasu Makuuchi

**Affiliations:** Department of Gastroenterological and General Surgery, Tokai University Hachioji Hospital, 1838 Ishikawacho, Hachioji City, Tokyo 192-0032 Japan

**Keywords:** Spontaneous mesenteric hematoma, Antithrombotic therapy, Acute discoid peritonitis, Intestinal failure, Surgical treatment, Sigmoid colon resection

## Abstract

**Background:**

Spontaneous mesenteric hematoma is a rare condition that is diagnosed when clinical and pathological findings do not identify an obvious causative disease. Various treatment options for spontaneous mesenteric hematoma exist; however, there are no clear treatment criteria. Herein, we report a case of spontaneous mesenteric hematoma that was successfully treated surgically and discuss the optimum treatment strategy based on similar cases.

**Case presentation:**

A 63-year-old man with abdominal persisting for 3 days presented to our hospital after going into shock without any triggers. The patient had a history of atrial fibrillation, stroke, and an aneurysm, and was receiving antithrombotic therapy. Abdominal contrast-enhanced computed tomography revealed a mass structure within the sigmoid mesentery, which was suspected to be a hematoma. The patient was admitted to the hospital for follow-up observation after initial infusion and vital stabilization. However, the following day, the patient developed acute generalized peritonitis with necrosis of the sigmoid colon; therefore, emergency Hartmann’s surgery was performed. Intraoperative and histopathological examinations revealed no evidence of bleeding.

**Conclusion:**

Spontaneous mesenteric hematomas tend to be associated with intestinal necrosis and may require surgical treatment with bowel resection owing to the difficulty in identifying the responsible vessel. Moreover, our results suggest that the presence of antithrombotic therapy may be an important factor affecting spontaneous mesenteric hematoma development.

## Background

Spontaneous mesenteric hematoma is a rare condition that is diagnosed when clinical and pathological findings do not identify an obvious causative disease [1, 2]. Treatment options include conservative, endovascular, and surgical treatments; however, there are currently no clear criteria for choosing a treatment course [3]. We encountered a case of spontaneous mesenteric hematoma that developed into peritonitis after conservative treatment had been chosen and was successfully treated surgically. Based on our own experience and previous case reports, we herein report on the tendency of this disease to develop and the decision regarding the optimum treatment strategy.

## Case presentation

A 63-year-old man with abdominal pain persisting for 3 days went into shock without any trigger, and was admitted to our hospital. The patient had been taking two antithrombotic drugs (cilostazol [200 mg; antiplatelet] and rivaroxaban [15 mg; direct oral anticoagulant]) owing to a history of multiple cardiovascular diseases, including atrial fibrillation, stroke, and left popliteal and internal iliac artery aneurysm. On arrival, the patient’s vital signs were as follows: a Japan Coma Scale (JCS) of 1, heart rate of 102/min, and blood pressure of 73/48 mmHg. The abdomen was tender from the pericardium to the lower abdomen; however, no symptoms of peritoneal irritation were noted and no traumatic scars were observed.

**Fig. 1:**
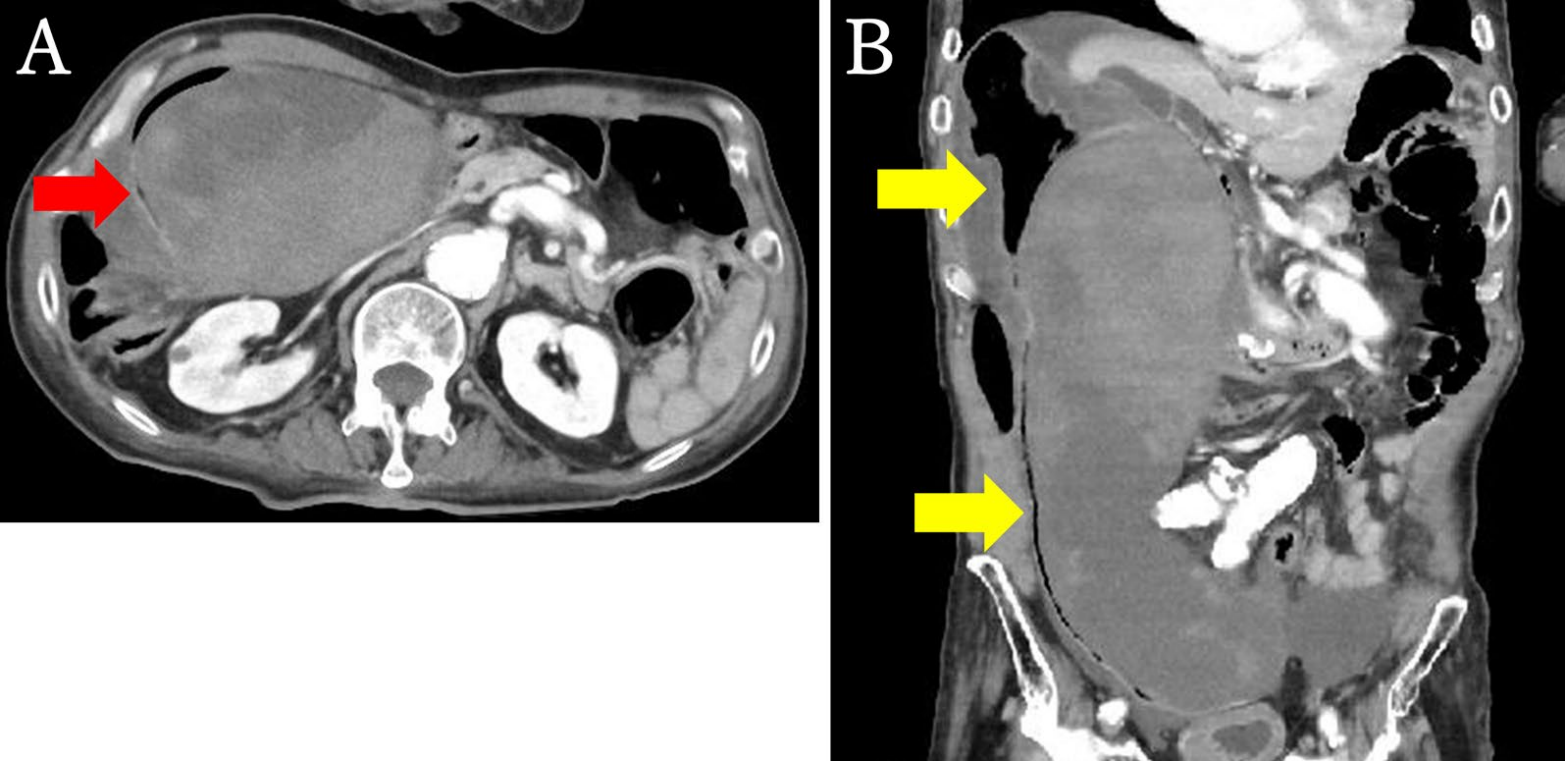
Abdominal contrast-enhanced CT scans. **A** Cross-sectional image (arterial phase). A 95-mm × 250-mm mass structure of the suspected mesenteric hematoma is observed along the sigmoid colon. The interior is accompanied by a heterogeneous hyperabsorptive zone (red arrow) and the arterial vessels are accompanied by severe atherosclerosis. **B** Coronal plane image (arterial phase). A mesenteric hematoma is observed along the sigmoid mesentery, compressing the sigmoid colon (yellow arrow). There is no obvious extra aneurysm in or around the hematoma and no free air in the abdominal cavity. CT: computed tomography

Blood test findings showed anemia, with a hemoglobin level of 9.0 g/dL, a C-reactive protein (CRP) level of 1.18 mg/dL, and elevated lactic acid at 5.7 mmol/L. However, other laboratory tests revealed no abnormalities. Contrast-enhanced computed tomography (CT) of the abdomen showed a 95-mm × 250-mm mass structure along the sigmoid colon with a suspected mesenteric hematoma. This was accompanied by a heterogeneous internal hyperabsorptive zone compressing the sigmoid colon. There was no obvious extravascular leakage of the contrast medium and identification of the responsible vessel was difficult. Moreover, no aneurysms or areas of intraabdominal free air were observed (Fig. 1A, B). Owing to the high risk of fatal complications due to the presence of multiple aneurysms and the absence of obvious extravascular leakage, endovascular treatment was not performed after consultation with the imaging department. The patient responded well to the initial transfusions and blood transfusions, and the vital signs were stable; therefore, the patient was admitted to the hospital for continuation of conservative treatment and follow-up.

**Fig. 2:**
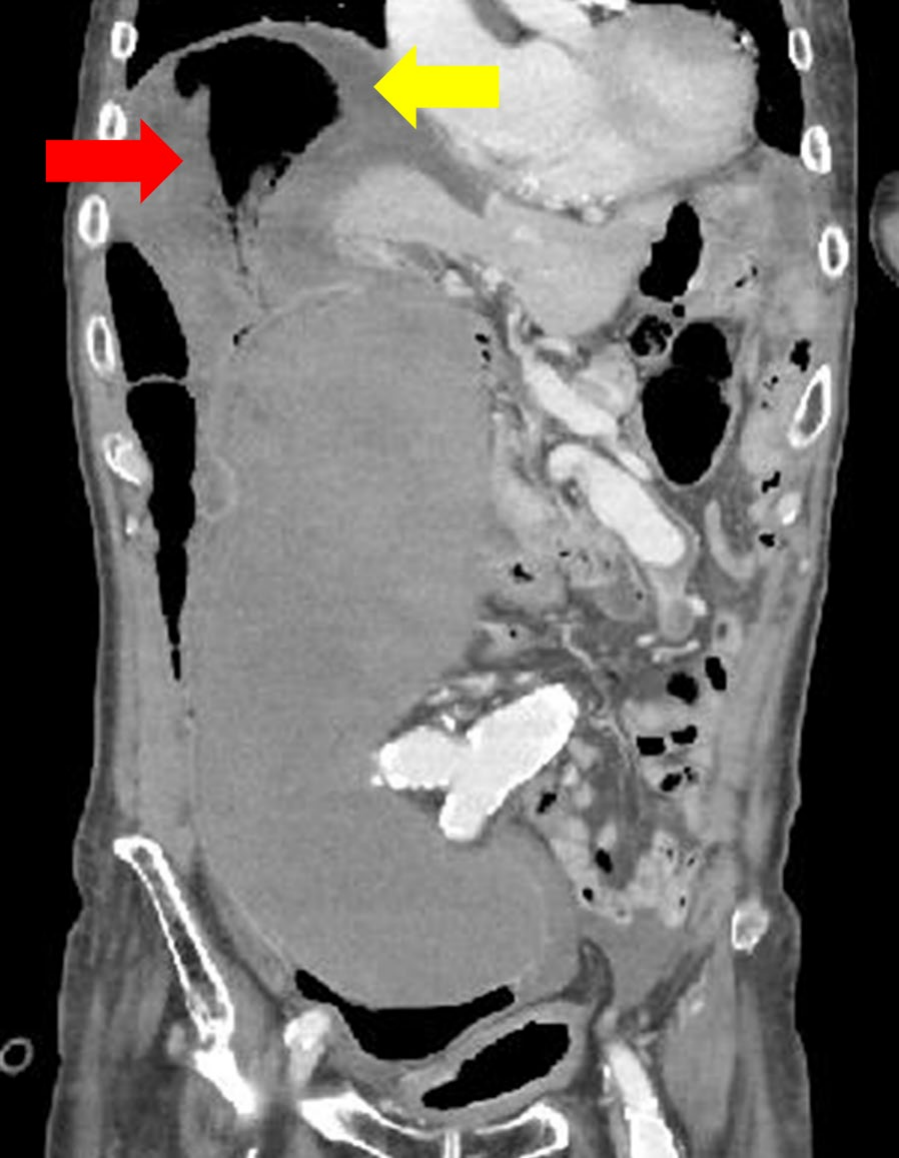
Abdominal contrast-enhanced CT scan on the first day in the hospital (equilibrium phase). The contrast effect on the sigmoid colon wall, compressed by the hematoma, is poor (red arrow) and the ascites are increased (yellow arrows). CT: computed tomography

On the first day in the hospital, peritoneal irritation signs appeared and blood test findings showed an increased inflammatory response, with a white blood cell count of 19,600/µL and a CRP level of 8.32 mg/dL. Therefore, another abdominal contrast-enhanced CT scan was performed. This showed no increase in hematoma or intraabdominal free air but revealed a poorly contrast-enhanced area of the sigmoid colon wall compressed by the hematoma and increased ascites (Fig. 2). Therefore, acute generalized peritonitis associated with necrosis of the sigmoid colon was suspected, and the patient was referred for emergency surgery.

**Fig. 3:**
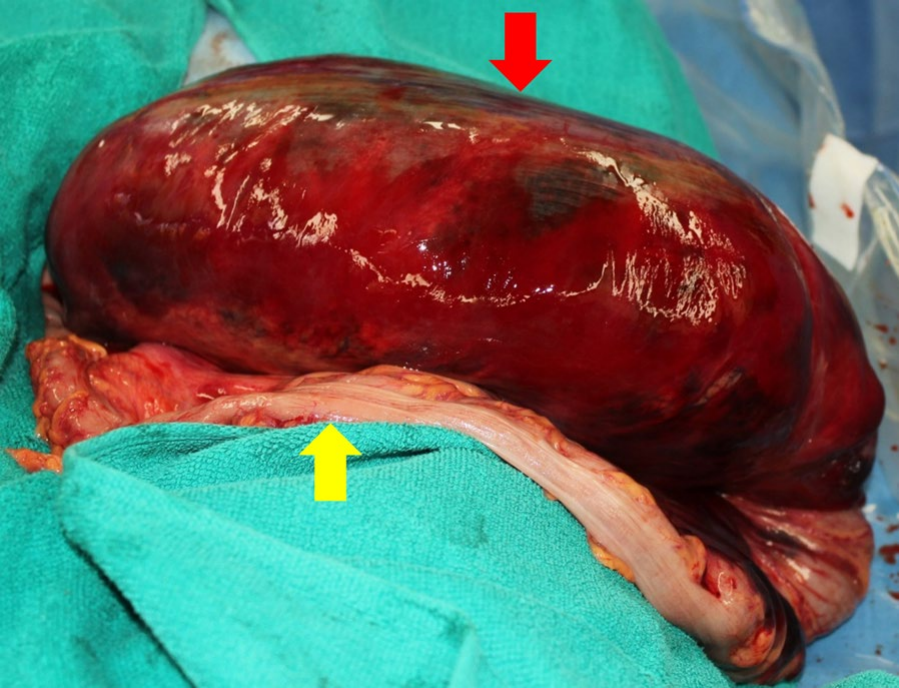
Operative findings. Image showing a massive hematoma in the sigmoid mesentery (red arrow). The sigmoid colon is compressed by the hematoma (yellow arrow)

**Fig. 4:**
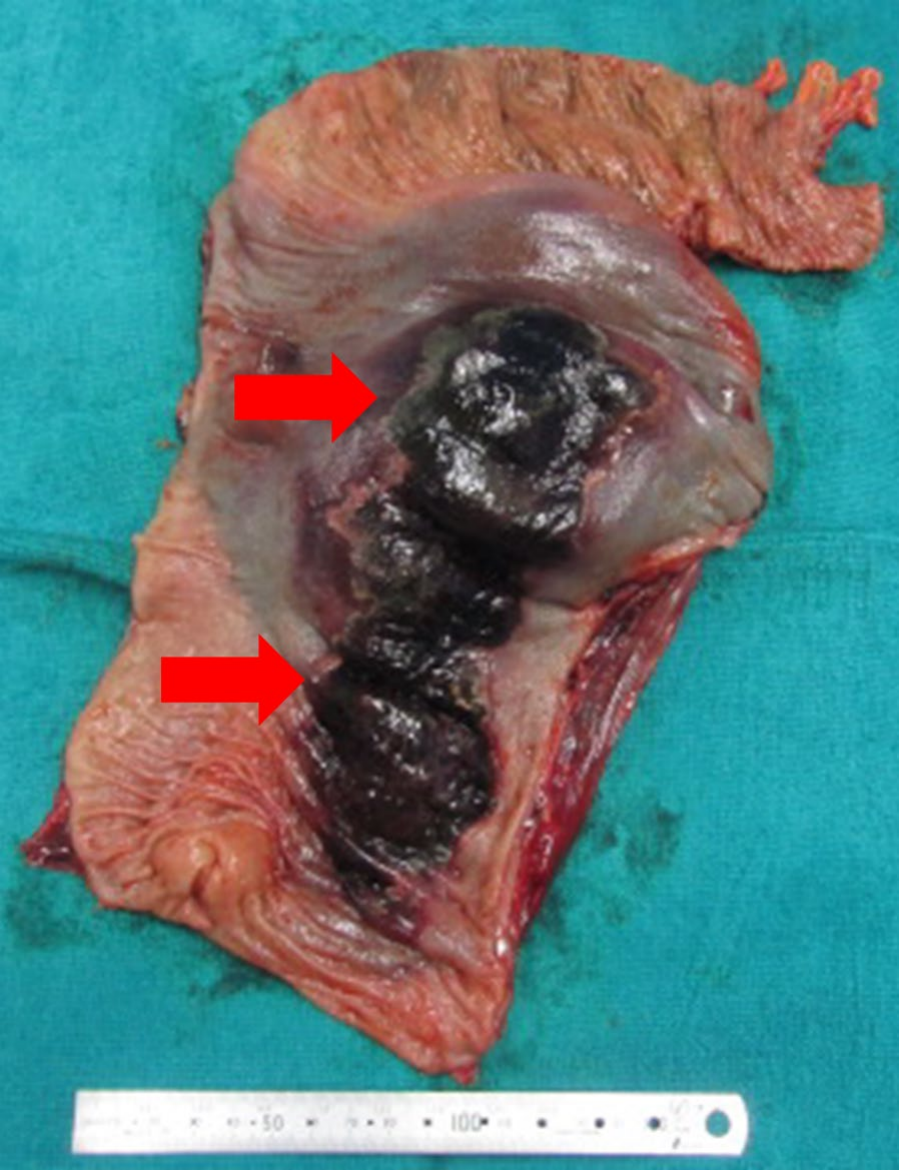
The excised specimen. The sigmoid colon is observed to be necrotic in all layers (red arrow). No perforation sites are observed

**Fig. 5:**
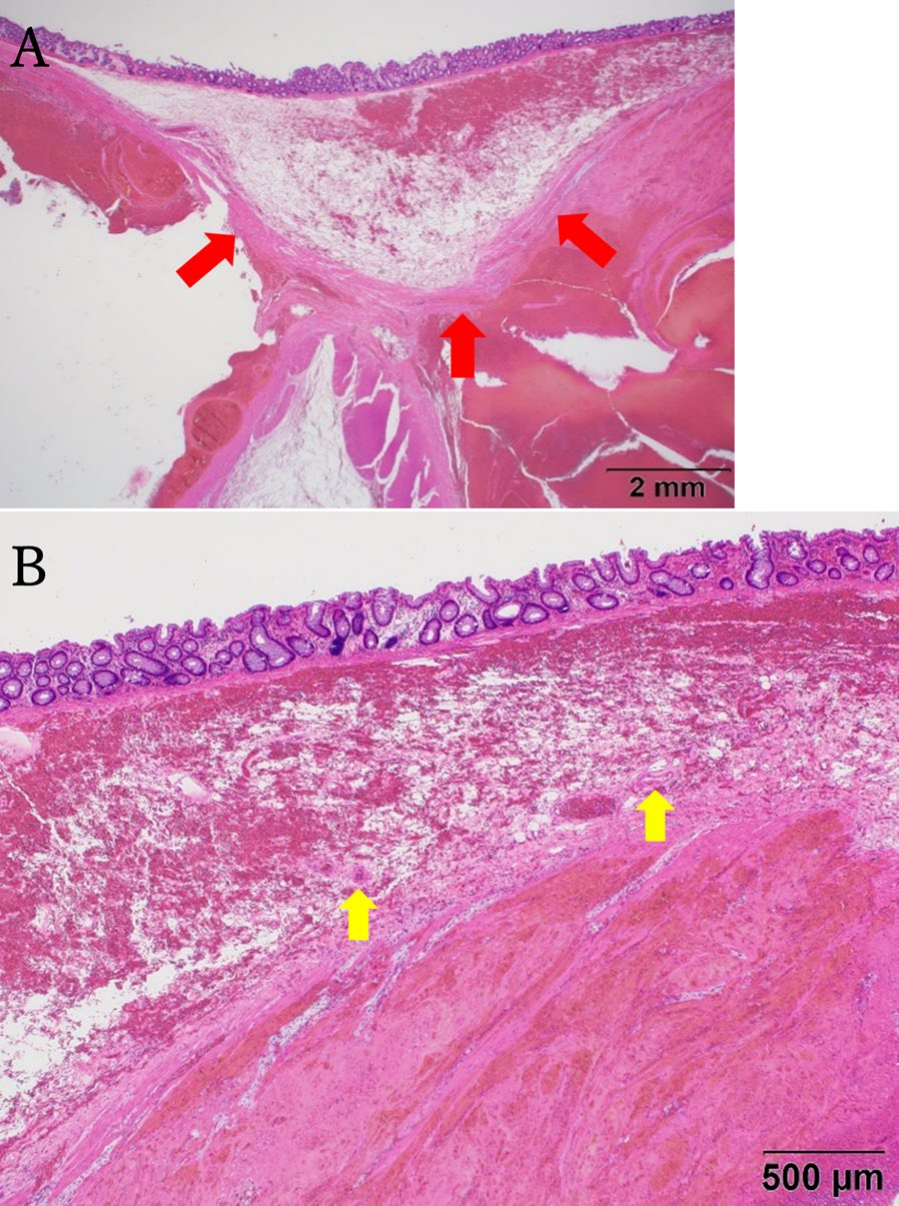
Pathological insights. **A** Upon dissection of the muscle layers, the hematoma is observed to be located between the intrinsic muscle layers (red arrow). **B** No obvious thrombus or vasculitis are observed in the vessels of the hematoma margins (yellow arrow)

The emergency surgery consisted of sigmoid colon resection and a single-hole descending colostomy. Surgical findings revealed a large hematoma contiguous with the sigmoid mesentery and a necrotic bowel of the sigmoid colon compressed by the hematoma (Fig. 3), with no observed contaminated ascites. Thereafter both the sigmoid colonic artery and the superior rectal artery were dissected, the hematoma and intestinal tract were removed as a single mass by dissection using a Powered ECHELON FLEX + GST System Green Cartridge (60 mm; J&J MedTech, New Brunswick, NJ, USA) at the sigmoid colon–descending colon junction on the oral side and the rectosigmoid junction on the anal side. However, the vessel responsible for the hemorrhage could not be identified. Finally, to complete the operation, a single-hole colostomy was created in the descending colon. Overall, the operative duration was 238 min, with a blood loss of 1072 mL (including the hematoma contents). Postoperatively, it was observed that the sigmoid mesentery was grossly enlarged by the hematoma, and the lumen of the colon was compressed by the hematoma. The sigmoid colon was necrotic in all layers on the mesenteric side of the resected specimen (Fig. 4). Histopathological findings showed that the hematoma extended from the mesentery to the submucosa. Although the hematoma was predominantly located between the intrinsic muscle layers in the colonic wall, the degree of damage was more severe on the mesenteric side. The normal structure of the mucosa of the colon and rectum was preserved (Fig. 5A). The vessels at the margins of the hematoma showed no obvious thrombi or vasculitis and no loss of elastic plates upon Elastica van Gieson staining (Fig. 5B). We diagnosed as a mesenteric hematoma based on gross and histopathological findings.

The patient showed a good postoperative course and oral drug intake was initiated on postoperative day 3. On postoperative day 4, the rivaroxaban dosage was reduced from 15 to 10 mg, and the two antithrombotic drugs were reinitiated. The patient was discharged from the hospital on postoperative day 7.

## Discussion

The pathogenesis of mesenteric hematoma involves local hemorrhage caused by the disruption of the mesenteric blood flow [3, 4], mostly due to traumatic or postoperative complications [1, 5]. In nontraumatic cases, an association with autoimmune diseases such as collagen diseases, vasculitis, and inflammatory bowel disease; degenerative diseases such as segmental arterial mediolysis; and genetic predispositions such as hemophilia, Ehlers–Danlos syndrome, and neurofibromatosis has been reported [6–12]. Spontaneous mesenteric hematoma is a rare disease that causes a hematoma in the mesentery without any clinicopathological findings such as trauma, autoimmune diseases, degenerative diseases, or genetic predisposition [1–3]. During diagnosis, differentiation of spontaneous mesenteric hematomas from ruptured aneurysms, which are highly lethal, is crucial; abdominal ultrasonography, contrast-enhanced CT, and magnetic resonance imaging are useful in this [3, 5, 11]. If a definitive diagnosis is made and the symptoms and hematoma size are minor, conservative treatment is possible [3, 13]. However, spontaneous mesenteric hematoma is a rare disease with nonspecific symptoms and imaging findings that change over time, making a definitive diagnosis difficult [4]. Surgery is indicated when a definitive diagnosis is challenging, when there is active bleeding with unstable vitals, or when symptoms caused by the hematoma are severe [11].

Although the patient had a history of multiple aneurysms, histopathological search, using hematoxylin eosin staining and Elastica van Gieson staining, for a mesenteric hematoma showed no inflammatory cell infiltration mainly based on macrophages and T lymphocytes or destruction of elastic fibers in the tunica media and adventitia of arteries, which would suggest aneurysm or aneurysm rupture. Also, the patient had a suspected but never diagnosed segmental arterial mediolysis and did not have the histopathological findings characteristic of segmental arterial mediolysis, such as vacuolization and lysis of the outer arterial media and loss of elastic lamina. The diagnosis of a spontaneous mesenteric hematoma was made as there were no explanatory clinical or pathological findings as to the cause of the hematoma. After the onset of abdominal pain, the patient had taken the antithrombotic drug for three days before being transported to our hospital. Antithrombotic drugs might have been a contributing and increasing factor to the spontaneous hematoma.

To explore the previous literature, we searched PubMed for "spontaneous mesenteric hematoma", "idiopathic spontaneous intraperitoneal hemorrhage/hematoma", and "spontaneous abdominal apoplexy." In total, 17 cases—including 17 spontaneous mesenteric hematomas reported over a 10-year period from 2013 to 2023 and an autopsy case—were identified (Table 1). The mean age at onset was 63.4 years, with a male-to-female ratio of 12:5. The first symptoms were abdominal pain in 76.4% (13 cases); nausea, vomiting, and anorexia in 23.5% (4 cases); diarrhea and blood loss in 11.8% (2); and abdominal distension in 6.9% (1 case), all of which were nonspecific symptoms. The dominant regions of hematoma origin were the superior mesenteric artery in 70.6% (12 cases), inferior mesenteric artery in 23.5% (4 cases), and unknown in 5.9% (1 case). Overall, the prognosis was good and no deaths were reported. These results were comparable to those reported by Yokota et al. and Kibe et al. in Japan, where reported cases accumulated and autopsy cases also had a relatively typical course [13, 14].

**Table 1:** Review of cases of idiopathic mesenteric hematomas reported in PubMed from 2013 to 2023 (including autopsy cases)

Authors	Age	Sex	Initial symptom	Location of hematoma	Maximum diameter (mm)	Responsible vessels	Hemorrhagic shock	Intestinal necrosis	Treatment	Antithrombotic therapy
Ros et al., 2014 [17]	71	M	–	Mesentery	120	–	〇	–	Conservative management	Oral anticoagulants
Traoré et al., 2016 [18]	35	F	Right basithoracic pain	Right colic angle	160	–	〇	–	Laparotomy	Enoxaparin–fluindione
Shikata et al., 2016 [2]	75	M	Anal bleeding Anemia	Jejunum	30	–	–	–	Mesenteric excision and partial enterectomy	–
Hirano et al., 2018 [1]	71	M	Abdominal pain Diarrhea	Sigmoid colon	100	Sigmoid colon artery	〇	〇	IVR + Hartmann	Rivaroxaban
Bekki et al., 2019 [19]	90	M	Lower abdominal pain	Transverse colectomy	90	–	–	–	A hand-assisted laparoscopic transverse colectomy	–
Nakamura et al., 2019 [20]	56	M	Lower abdominal pain	Transverse colectomy	130	Middle colic artery	〇	–	Ligation of responsible vessels	–
Montero et al., 2019 [21]	67	F	Abdominal pain	Ileum	–	–	–	〇	Partial enterectomy	Warfarin
Negmadjanov et al., 2019 [22]	51	F	Abdominal pain Abdominal distention	Mesentery	150	Middle colic artery	〇	–	Ligation of responsible vessels	–
Vecchio et al., 2019 [5]	48	M	Thoracic-abdominal pain Fever	Caecum ascending colon	–	–	–	〇	Right hemicolectomy	–
Tanioka et al., 2020 [4]	69	M	–	Jejunum	75	–	–	–	Mesenteric excision and partial enterectomy	–
Qaraqe et al., 2021 [23]	27	M	Abdominal pain vomiting	Transverse colon	–	Middle colic artery	〇	〇	Partial transverse colectomy	–
Urabe et al., 2021 [24]	81	M	–	Descending colon	33	–	–	–	Descending colectomy	Aspirin
Phua et al., 2021 [15]	60	F	Abdominal pain	Jejunum ~ ileum	220	Superior mesenteric artery	〇	–	IVR	Rivaroxaban
Phua et al., 2021 [15]	83	F	Abdominal pain	Transverse colon ~ Descending colon	–	–	〇	〇	Partial colectomy and splenectomy	Apixaban
Alyaseen et al., 2022 [3]	45	M	Abdominal pain vomiting	Jejunum ~ ileum	150	Superior mesenteric artery	〇	–	Ligation of responsible vessels	–
Wu et al., 2022 [25]	87	M	Abdominal pain anorexia	Jejunum	–	–	–	–	Conservative treatment	Acenocoumarol
Present case	63	M	Abdominal pain	Sigmoid colon	250	–	〇	〇	Hartmann	Cilostazol Rivaroxaban

F: female, M: male

To our knowledge, there is no well-known and clear protocol for the treatment of spontaneous mesenteric hematomas. Therefore, conservative, endovascular, and surgical treatments should be considered individually based on imaging findings and the clinical course. According to reports by Phua et al., arterial embolization may be a minimally invasive and reliable method for small hematomas with a stable general condition (shock index of 0.5–0.9) if the responsible vessel can be identified. [15]. However, Parker et al. found that in approximately one-third of spontaneous mesenteric hematomas, the source of the hemorrhage cannot be identified and endovascular treatment has limitations because samples of the vessel wall and surrounding tissue cannot be taken and a histopathological diagnosis cannot be reached; therefore, a careful decision regarding the indication for treatment is necessary [11]. In contrast, Abe et al. reported that surgical treatment with hematoma removal and bowel resection to ensure removal of the causative site is necessary when intestinal necrosis is observed or when the cause of bleeding is unknown and rebleeding is possible [16]. In our literature search, we found that endovascular and surgical treatments were performed in 11.8% (2 cases) and 82.4% (14 cases) of cases, respectively, with 78.6% (11/14 cases) of surgical treatments involving bowel resection in addition to hematoma removal. The responsible vessel was unknown in 64.7% (11 cases) of the cases. If a hematoma near the colon compresses the colon, it can cause necrosis and perforation of the colon wall, and obstruction of the lumen. In addition, intestinal obstruction and peritonitis may occur. In the present study, 33.3% (6 cases), including the autopsy cases, developed intestinal obstruction or peritonitis. Typically, those complications can be fatal due to delayed treatment, and early consideration of surgical bowel resection including hematoma in patients with mesenteric hematomas can be helpful. Based on their pathogenesis, the distance between the hematoma and the intestinal tract and the size of the hematoma may be associated with the incidence of complications, but previous reports have not always specified the respective values, making it difficult to investigate correlations. Further case accumulation is desirable.

These results suggest that surgical treatment is effective for spontaneous mesenteric hematomas, and that it is particularly important to select a surgical technique considering intestinal necrosis. In our case, endovascular treatment was not indicated because the patient had a history of multiple aneurysms and a background disease such as segmental arterial mediolysis causing vessel wall fragility was suspected. Moreover, the hematoma was massive and the responsible vessel was unknown upon preoperative examination. The patient eventually developed acute generalized peritonitis due to bowel necrosis and was successfully treated surgically with bowel resection.

Importantly, we found that in our case, as well as in 52.9% (9 cases) of previous cases, antithrombotic therapy was present, which may be a risk factor for the development of spontaneous mesenteric hematoma. Therefore, spontaneous mesenteric hematoma should be considered in the differential diagnosis for acute abdomen in patients receiving antithrombotic therapy.

## Conclusions

Spontaneous mesenteric hematoma is a rare disease for which a definitive diagnosis is difficult. The disease should be considered as a differential diagnosis for acute abdomen, especially if the patient is on antithrombotic medication. We conclude that because intestinal necrosis may occur during the course of the disease and the responsible vessel is difficult to identify, bowel resection—including the hematoma—should be considered as a surgical treatment.

## Data Availability

The authors declare that all the data are available from the corresponding author upon request.

## Abbreviations

JCS: Japan Coma Scale
CT: Computed tomography
CRP: C-reactive protein
